# The association between body mass index and the risk of different urinary cancers: Protocol for an overview of systematic reviews

**DOI:** 10.1097/MD.0000000000021362

**Published:** 2020-07-24

**Authors:** Wenli Zhao, Jiyuan Shi, Yamin Chen, Ziwei Song, Liangliang Si, Xin Jiang, Yu Gu

**Affiliations:** a Catheter Lab, Henan Provincial People's Hospital, and People's Hospital of Zhengzhou University, Henan University People's Hospital, Zhengzhou City; b Evidence-Based Nursing Centre, School of nursing, Lanzhou University, Lanzhou City, Gansu Province, China.

**Keywords:** cancer, meta-analysis, obesity, overweight

## Abstract

**Background:**

The relationship between cancer with body mass index has been extensively reported. However, association between urinary cancers with these risk factors remains unclear, with existing reports showing conflicting findings. The current review, therefore, sought to clarify the latter association by assessing the methodological and reporting quality of existing systematic reviews on the subject.

**Methods:**

We will screen PubMed, EMBASE, and Cochrane Library databases for relevant literature and subjected the resulting articles to meta-analysis. We will adopt the AMSTAR and PRISMA checklists for assessing methodological, and reporting quality, respectively. The association between BMI and different urinary cancers will be estimated by computing the pooled relative risk (RR) and its 95% confidence interval (CI), which will be calculated from the adjusted RR, odds ratio, or hazard ratio, and 95% CI offered in the studies. Heterogeneity between studies will be assessed with the I statistic as a measure of the proportion of total variation in estimates that is due to heterogeneity, where I values of 25%, 50%, and 75% correspond to cut-off points for low, moderate, and high degrees of heterogeneity. The random effects model will be used as the pooling method when significant heterogeneity existed and the fixed effect model will be used when no heterogeneity was observed. Possible publication bias will be tested by Begg and Egger test.

**Conclusion:**

Our evidence synthesis will provide a new commentary on the current systematic review evidence for the association between BMI and the risk of different urinary cancers.

**PROSPERO registration number:**

CRD42019119459.

## 1 Introduction

Cancer is the second most deadly disease affecting human health worldwide.^[1]^ According to the Global Cancer Statistics of 2018, published by the World Health Organization/International Agency for Research on Cancer,^[2]^ prostate cancer represents the second most common type of cancer and the fifth leading cause of cancer-related deaths in men. For example, this type caused an estimated 1.3 million new cases and 359,000 deaths in 2018 alone, whereas bladder cancer accounted for 549,000 new cases and 20 million deaths worldwide. The incidence of bladder cancer (9.6/100,000) as well as mortality rate (3.2/100,000) are about four times (2.4/100,000 and 0.87/100,000) that of females. On the other hand, kidney cancer caused >400,000 new cases, and 170,000 deaths in the same year.^[2]^ These figures underscore the incidence and impact caused by the three types of cancers across the world,^[2,3]^ although the underlying mechanism of their development remains unclear owing to limited evidence. Previous studies have suggested that cumulative effects of cigarette smoking, alcohol consumption, obesity, and genetic susceptibility may be risk factors for urinary cancer.^[3,4]^ Accurate understanding of these risk factors is critical for the development of effective approaches for cancer prevention and treatment.

Overweight and obesity are defined as excess body weight that causes many chronic diseases and increases the risk of death. The number of overweight and obese adults had risen to 2.1 billion in 2013, with direct costs resulting from obesity estimated to account for 0.7 to 2.8% of a country's total healthcare expenditures.^[5,6]^ In USA, Wang et al^[7]^ predicted a $48 to $66 billion increase per year in combined medical costs from common obesity-related diseases by 2030. Policymakers in the public health sector rely on high-quality evidence, generated by meta-analyses and SR, to formulate policies for the prevention and management of cancer. However, despite numerous SRs describing the relationship between cancer with overweight and obesity, over the past several decades, conflicting findings regarding the association of these risks factors with urinary cancers pose a challenge to accurate understanding of their epidemiology as well as the development of management approaches.^[8–12]^

In the current study, we sought to generate more comprehensive and robust evidence of the relationship between the urinary cancers with obesity and overweight using a meta-analysis of published literature.

## 2 Material and methods

### 2.1 Protocol registration

This overview was registered by the International prospective register of systematic reviews (PROSPERO), number CRD42019119459.

### 2.2 Searching

Two reviewers will independently search the PubMed, Cochrane Library, and Embase databases. The search was limited to articles written in English and used the following search terms: BMI, obesity, cancer, carcinoma, neoplasm, meta-analysis, and SRs. A detailed description of the PubMed search strategy is presented in the Data S1.

### 2.3 Screening and selection procedure

Studies will be included if:

(1) SRs or meta-analysis associated obesity, and overweight with urinary cancer;
(2) SRs or meta-analysis associated increase in BMI with urinary cancer;
(3) articles were published in English;
(4) latest article was included when SRs or meta-analysis had been updated.

Studies were excluded if:

(1) they were only abstracts and/or letters;
(2) SRs and meta-analysis examined the association between BMI increase and prognosis, survival or recurrence of urinary cancers;
(3) protocols of SRs and meta-analysis or methodological articles;
(4) SRs without meta-analysis.

### 2.4 Study selection and data retrieval

The retrieved articles were first imported into EndNote X7 software, then titles and abstracts independently selected by two reviewers. The reviewers thereafter retrieved full texts of potentially eligible studies and independently subjected them to the aforementioned criteria. Any disagreement was discussed with a third reviewer. The 2 reviewers independently extracted the following characteristic from each study: first author's name, year of publication, funding, number of reference test, name of database, country of first author, the epidemiological study design (case-control or cohort), number of cases, features of the urinary cancers, summary effects between BMI and cancer risk (at 95% CI), and the number of included studies.

### 2.5 Methodological and reporting quality assessment

To assess methodological quality, we will use the AMSTAR checklist, which comprises 11 items. Total AMSTAR scores will be denoted as 1 point for “Yes” (item/question fully solved), no point for “No” (item/question not solved), or “partial answer” (item not fully solved). Obvious, moderate and minimal flaws will be defined using <4, 5–8, and <9 points, respectively as previously described.^[13,14]^ To assess the reporting quality of included SRs and meta-analyses, we will use the PRISMA checklist, which comprises 27 items. To show the degree of compliance, total PRISMA scores will be calculated by summing 1 point for “Yes” (total confirmed), 0.5 points for “Partial” (partial confirmed) and “Cannot answer” (limited information), 0 point for “No” (noncompliance).^[15]^ The SR and meta-analysis will be regarded low quality if PRISMA scores are below 15 points, moderate if they recorded 15.0 to 21.0 points, and high if >21.0 points are recorded.^[14,16]^ Quality assessment of the included SRs and meta-analyses will be independently performed by two authors (JYS and LLS), and any disagreements between them will be discussed with a third author (JHT).

### 2.6 Statistical analysis

We will calculate pooled RRs (at 95% CI) from included SRs and meta-analysis (HRs and ORs equivalent to RR) records using the fixed effects model or random-effects model.^[17]^ A condition is considered normal if a BMI of 18.5–24.9 kg/m^2^ was recorded, overweight for 25 to 29.9 kg/m^2^, and obesity for BMI ≥ 30 kg/m^2^. We will also analyze each 1 kg/m^2^ and 5 kg/m^2^ increase in BMI according to previous protocols.^[18,19]^ In addition, we will assesses the heterogeneity between studies using the I^2^ statistic.^[20]^ Specifically, we will adopt the fixed-effect model when I^2^ value was less than 50%, and the random-effect model for an I^2^ value greater than 50%.^[21]^ Possible publication bias will be tested by Begg and Egger test.^[21]^ Furthermore, we will analyze indirect comparisons of the outcomes across the meta-analyses. Statistical analyses will be performed using STATA software (version 12.0, College Station, TX), with values that have *P* ≤ .05 considered statistically significant. Bubble diagram will be constructed to visualize the methodological quality and the quality of included literature.

## 3 Discussion

Methodological and reporting quality of meta-analysis and SRs are crucial to public health and clinical decision making. Despite numerous studies analyzing cancer risk factors, no consensus has been reached regarding the relationship between different urinary cancer with obesity and overweight. In the current study, we sought to generate more comprehensive and robust evidence of the relationship between the urinary cancers with obesity and overweight using a meta-analysis of published literature.

## Acknowledgments

We express our thanks to Jinhui Tian for the assistance with the design of the protocol.

## Author contributions

JS and LLS conceived the idea for this study; JS, XJ, YG, and WLZ drafted the protocol. All authors approved the article in its final form.

**Writing – original draft:** Jiyuan Shi.
